# An Unexpected Finding of an Aggressive Pleural Hemangiopericytoma/Solitary Fibrous Tumor in a Patient With Recurrent Meningioma: A Case Report

**DOI:** 10.7759/cureus.54965

**Published:** 2024-02-26

**Authors:** Shahir Basir, Jana Bosiers, Nico C van Walree

**Affiliations:** 1 Respiratory Medicine, Antwerp University Hospital, Antwerp, BEL; 2 Respiratory Medicine, Amphia Hospital, Breda, NLD

**Keywords:** solitary fibrous pleural tumor, malignant diseases, nab2-stat6, primary pleural tumors, hemangiopericytoma

## Abstract

Pleural hemangiopericytoma/solitary fibrous tumor (HPC/SFT) is a rare form of mesenchymal tumor arising from pericytes, which predominantly occurs intrathoracically. HPC/SFT can be suspected on imaging, but radiographic features are non-specific. Therefore, histological confirmation remains the gold standard. Due to the rarity of the tumor, specific anatomical pathological expertise is necessary to make the diagnosis, which is not available in every hospital.

Here, we report the case of a 51-year-old female with a medical history of recurrent meningiomas. A chest CT scan revealed extensive subpleural soft tissue lesions in the left hemithorax with histological characteristics suggestive of a pleural malignancy. A specialized analysis of the sample led to the final diagnosis of HPC/SFT. Unfortunately, in the meantime, the patient’s condition worsened rapidly, and she passed away before the final diagnosis was made and any decisions about therapeutic options were taken. In our case, we want to highlight the importance of having knowledge about the existence of this type of tumor in order to make the correct diagnosis in a timely manner.

## Introduction

Pleural hemangiopericytoma/solitary fibrous tumor (HPC/SFT) is a rare form of mesenchymal tumor that arises from pericytes and accounts for <2% of all soft tissue tumors. HPC/SFT may be found anywhere in the body with a predominant intrathoracic occurrence. The most frequent thoracic localization of an HPC/SFT is the pleura, but in general, it accounts for <5% of all pleural tumors [1,2]. HPC/SFTs are mostly accidental radiological findings and have no specific radiological features. The diagnosis and treatment of this type of tumor remain challenging as they can mimic many different tumors on imaging. In the past, HPC/SFT has been successively referred to as localized mesothelioma, localized fibrous tumor, fibrous mesothelioma, or pleural fibroma, due to controversies regarding its histogenesis [1-3]. Specialized pathological analysis is necessary to be able to make the diagnosis and start the patient on the correct therapy [3]. Here, we report a rare case of an HPC/SFT of the pleura in a patient with recurrent meningiomas.

## Case presentation

A 51-year-old female presented to the emergency department with progressive dyspnea and a productive cough. In her medical history, recurrent meningioma with secondary symptomatic epilepsy was retained. On clinical examination, a complete care-dependent patient with tachypnea (24 times per minute), a saturation of 99% on ambient air, tachycardia (112 beats per minute {bpm}), and normal blood pressure and temperature was seen. The auscultation of the lungs revealed the complete attenuation of lung sounds over the left lung with dull percussion. Furthermore, several slow-growing nodules at the level of the scalp were observed, which were thought to be either keloid or recurrent meningioma.

**Figure 1:**
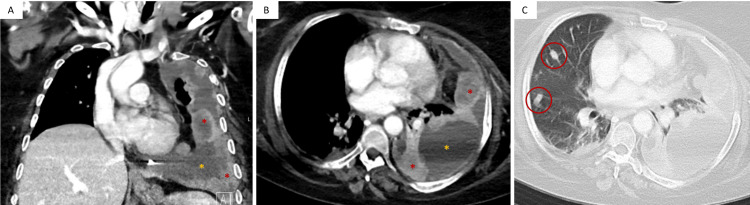
Contrast-enhanced CT scan of the chest revealing a large left-sided pleural mass consistent with a hemangiopericytoma/solitary fibrous tumor. Combined sagittal (A) and axial (B) views in the mediastinal setting and axial (C) view in the lung setting of the chest CT scan, showing a complete opacification of the left hemithorax with a contralateral mediastinal shift. The CT scan also showed extensive (>10 cm) subpleural soft tissue lesions in the left hemithorax (red asterisk) together with a significant amount of pleural fluid (yellow asterisk) and a complete atelectasis of the left lung, as well as multiple lung nodules (red circle) on the right side.

A chest CT scan showed the complete opacification of the left hemithorax with a contralateral mediastinal shift (Figure 1). Additionally, the CT scan revealed extensive subpleural soft tissue lesions in the left hemithorax together with a significant amount of pleural fluid and a complete atelectasis of the left lung. Multiple lung nodules were also present on the right side.

Thoracocentesis was performed yielding 500 mL of clear, yellow, serous fluid. The analysis of this pleural fluid was consistent with an exudative pleural effusion (protein, 35 g/L; lactate dehydrogenase {LDH}, 903 U/L; glucose, 6.1 mmol/L; and pH, 7.4). Gram staining and cultures were negative. Cytological analysis revealed that the fluid was rich in lymphocytes, but no atypical cells were present. Because of the rapid recurrence of pleural fluid together with respiratory insufficiency, a CT-guided pleural biopsy was performed, and a chest drain was placed.

**Figure 2:**
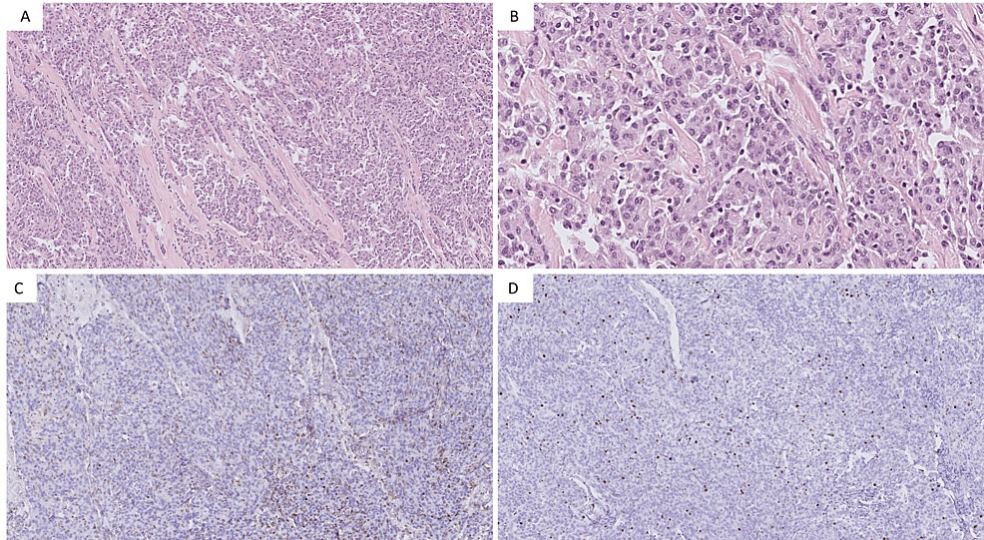
Hematoxylin and eosin (H&E) staining and immunohistochemical staining of the pleural mass. (A) H&E staining of 5× magnification and (B) H&E staining of 20× magnification of the tumor, showing atypical spindle cell proliferation; (C) 10× magnification of the tumor with Bcl-2 immunohistochemical staining that is positive; and (D) 10× magnification of the tumor with MIB-1 immunohistochemical staining that is positive. Bcl-2, B-cell lymphoma 2; MIB-1, mindbomb E3 ubiquitin protein ligase 1

The histological examination of the pleural biopsy revealed an atypical spindle cell proliferation of malignant origin (Figure 2). Given the female’s medical history and the current presence of suspicious lesions on her scalp, the differential diagnosis of metastasis of her meningioma was considered. More specialized analysis of the sample in a university pathological laboratory was required and led to the final diagnosis of an HPC/SFT. Unfortunately, in the meantime, the patient’s condition worsened rapidly. She developed respiratory failure due to the recurrent production of massive amounts of pleural fluid, which could not be alleviated with repeated thoracentesis. The patient passed away before the final diagnosis was made and any decisions about therapeutic options could be taken.

## Discussion

Pleural HPC/SFT is a rare form of mesenchymal tumor/sarcoma arising from pericytes [1,2]. HPC/SFTs make up approximately 2% of all soft tissue sarcomas in the body. HPC/SFTs of the pleura are relatively rare with an incidence of <3 per 100,000 patients and account for <5% of all pleural tumors. Pleural HPC/SFTs occur mainly in adults at the age of 40-70 years and affect an equal number of males and females [1,2]. Usually, an HPC/SFT of the pleura presents as a solitary, localized mass with a smooth surface, attached to the pleura with a vascularized pedicle. Up to 80% of HPC/SFTs can occur from the visceral pleura [4], with a slight predominance of pedunculated tumors. Often, they are localized in the inferior hemithorax [5,6]. A diameter greater than 10 cm is usually associated with malignancy [7]. In our case, a large, irregular pleural abnormality was seen, suspicious for a pleural malignancy. An HPC/SFT can thus be suspected on imaging, but radiographic features are non-specific [1,2]. Therefore, histological confirmation remains the gold standard in the diagnosis of HPC/SFT [3].

Based on histological features, HPC and SFT were previously considered to be two separate entities. However, the discovery of *NAB2-STAT6* fusion as a specific molecular alteration in both tumors led to a combined reclassification in the 2016 WHO classification of central nervous system tumors, distinguishing three histological grades [1-3]: (i) Grade 1 tumors have high collagen content and low cellularity with spindle-like cells; these tumors are generally considered benign and were previously referred to as SFT. (ii) Grade 2 tumors contain less collagen and have higher cellularity with a pattern-less architecture surrounded by staghorn vasculature; previously, these tumors were referred to as HPC. (iii) Grade 3 tumors are those previously called anaplastic HPC, characterized by >5 mitoses per 10 high-power fields.

Moreover, HPC/SFT is histologically characterized by a multiplicity of growth patterns. Two major histological growth patterns of pleural HPC/SFT have been described: (i) diffuse sclerosing and (ii) solid spindle, with admixing of varying proportions. Solid spindle patterns are characterized by a histological appearance that varies from area to area within the tumor. The most frequent solid spindle pattern is the short storiform, which is characterized by an oval-to-spindle pattern showing variable short storiform of cartwheel formations. Hemangiopericytoma-like growth (staghorn) is the second most common subtype of the solid spindle patterns [7]. In our case, the histological analysis of the tumor showed the proliferation of spindle-shaped cells with a storiform pattern. The spindle-shaped cells showed partly slender and partly plump nuclei with slightly irregular core contours. Occasional mitotic spindle figures were observed. In addition, the cells partly extended into the alveolar lung parenchyma that was present in the studied biopsy sections. Based on these histological findings, together with the patient’s medical history, the tumor was initially thought to be a pleural metastasis of her meningioma, but the diagnosis of an HPC/SFT was considered as well since HPC/SFT can radiologically and histologically closely resemble a meningioma. Specific anatomopathological expertise was needed to come to a final diagnosis. As this expertise was not available in our hospital, the biopsy specimen was sent to a university pathological laboratory for such specialized immunohistochemical analysis.

Immunohistochemically, HPC/SFTs are typically positive for cluster of differentiation (CD) 34 (sensitivity of 96%) and often also stain positive for B-cell lymphoma 2 (Bcl-2) (sensitivity of 94%) and CD99 (sensitivity of 88%). Calretinin, S-100, desmin, smooth muscle actin, actin, neurofilament protein, and epithelial membrane antigen (EMA) on the other hand are usually negative in HPC/SFTs [8]. In a minority of cases, there is a lack of CD34 expression in the HPC/SFT. The latter appears to be a characteristic of the more malignant forms of HPC/SFT [9]. Additionally, multivariable analysis showed that both a tumor size of ≥10 cm and a high mindbomb E3 ubiquitin protein ligase 1 (MIB-1) proliferation were independently associated with adverse outcomes [10,11]. In our case, immunohistochemical staining was negative for CD34 but positive for Bcl-2. In addition, calretinin and EMA expressions were also examined, and both were found to be negative. Therefore, overall, the immunohistochemical pattern favored the diagnosis of an HPC/SFT tumor but with malignant characteristics because of the CD34 negativity of the pleural tumor.

The treatment of HPC/SFTs consists of surgical (radical) resection, with or without adjuvant radiotherapy and/or chemotherapy. Adjuvant radiotherapy remains a point of discussion as there is a proven effect on local disease control described in the literature, but no metastasis prevention has been observed. Conventional chemotherapy provides limited clinical benefit. Anti-angiogenetic therapy appears to be promising but is currently only used as a third-line treatment in selected patients [3]. The majority of HPC/SFTs behave in an indolent manner, with the 10-year survival rates for both pleural and extrapleural HPC/SFTs between 54% and 89% after resection [12].

In our case, the clinical condition of the patient deteriorated rapidly, and she passed away before the final pathological diagnosis was made and therapy could be started. Although most HPC/SFTs behave indolently and have a favorable outcome, this was not the case in our patient. In retrospect, the patient’s rapid clinical deterioration can most likely be explained by the fact that she had an HPC/SFT with unfavorable characteristics, including a tumor size of >10 cm on CT of the thorax, a lack of CD34 expression, and high MIB-1 positivity.

## Conclusions

Here, we report a rare case of an HPC/SFT of the pleura, which is a rare form of a mesenchymal tumor that does not have typical radiological features and can mimic different types of tumors. This kind of tumor is infrequently encountered by pneumologists and poses a diagnostic challenge. With this case, we want to draw the attention that it is important to have at least a basic knowledge of the existence of this type of tumor and to ask for specialized analysis promptly to be able to start the patient on the correct therapy as soon as possible.
